# Polio Amidst COVID-19 in Pakistan: What are the Efforts Being Made and Challenges at Hand?

**DOI:** 10.4269/ajtmh.20-1438

**Published:** 2020-12-02

**Authors:** Shoaib Ahmad, Maryam Salma Babar, Attaullah Ahmadi, Mohammad Yasir Essar, Uzzam Ahmed Khawaja, Don Eliseo Lucero-Prisno

**Affiliations:** 1 Punjab Medical College, Faisalabad, Pakistan;; 2 Dubai Medical College, Dubai, United Arab Emirates;; 3 Medical Research Center, Kateb University, Kabul, Afghanistan;; 4 Kabul University of Medical Sciences, Kabul, Afghanistan;; 5 Jinnah Medical and Dental College, Karachi, Pakistan;; 6 Department of Global Health and Development, London School of Hygiene and Tropical Medicine, London, United Kingdom;; 7 Faculty of Management and Development Studies, University of the Philippines (Open University), Los Baños, Laguna, Philippines

## Abstract

Poliomyelitis, a crippling viral disease, has been affecting many children in Pakistan despite the numerous efforts that have been taken to curb its spread. The COVID-19 pandemic has halted mass polio vaccination campaigns globally, including Pakistan, resulting in a resurgence of new cases. Pakistan managed to flatten the COVID-19 curve from July to October that made three immunization drives possible, but the COVID-19 cases are on the rise again which can again complicate the polio situation in the country if left unmonitored. The efforts of Pakistan have been effective with no significant rise in polio cases in 2020 as compared with 2019. We discuss the numerous challenges faced by the polio eradication program in Pakistan. To help eliminate polio, Pakistan needs to enhance its efforts in the struggle against polio with the same zeal and stringency used to flatten the curve of COVID-19 in these challenging times.

## INTRODUCTION

Poliomyelitis, a highly infectious and crippling disease caused by the poliovirus is widely known to affect unimmunized children. Person-to-person transmission of the virus occurs through the fecal–oral route. To date, there is no treatment available for polio, but the disease can easily be prevented by vaccine administration. As 90% of infected individuals do not display any symptoms, they can easily go undetected, but are carriers of the virus. Mass vaccination prevents community transmission. Since 1988, there has been a 99% decline in cases of polio worldwide,^1^ and there are now only two endemic countries, Pakistan and Afghanistan, proving the success of a worldwide vaccination program.^2^

The Global Polio Eradication Initiative (GPEI) is a public–private partnership comprising six organizations, namely, the WHO, CDC, UNICEF, Rotary International, Bill & Melinda Gates Foundation, and Gavi, the Vaccine Alliance. The GPEI is working to eradicate polio from the globe. However, to shift resources to the battle against COVID-19, the GPEI suspended its polio eradication program until the second half of 2020. This suspension was accompanied by a resurgence of polio cases in Afghanistan in 2020.^2^

**Figure 1.**
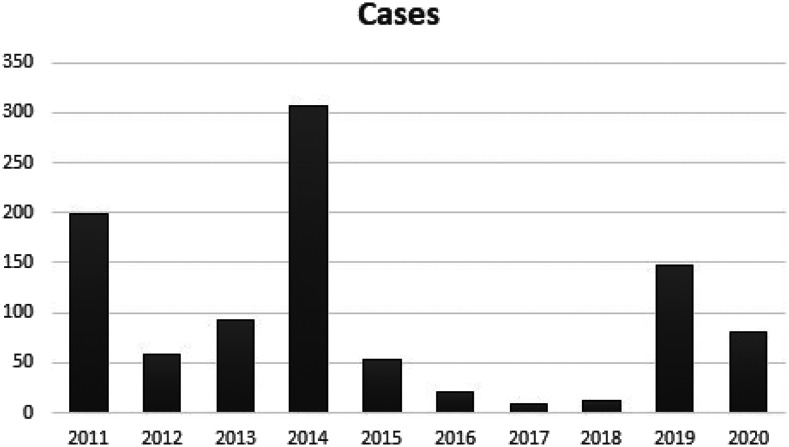
Wild poliovirus cases in Pakistan in the last decade as of November 16, 2020. (Source: https://www.endpolio.com.pk/media-room/pakistan-polio-update)

Pakistan, against all odds, managed to flatten the COVID-19 curve, keeping the positivity rate of tests to less than 2% for most of July, August, September, and October. Hence, it resulted in better focus on other epidemics such as polio, and successful drives were carried out.^3^ As of November 16, 2020, 81 polio cases have been reported in Pakistan, compared with a total of 147 cases in all of 2019 (Figure 1).^4^

## EFFORTS BEING MADE

The WHO reported that approximately 80 million vaccination opportunities were missed by Pakistani children.^5^ In wake of this, after a 4-month hiatus (end of March–mid July) and the emergence of new polio cases, a small-scale polio vaccination drive took place at the end of July 2020, reaching about 780,000 children.

As of August 11, 2020, Pakistan had resumed mass vaccination campaigns in the hopes of halting a polio outbreak, which would overburden an already struggling health system. In August 2020, a 3-day national anti-polio campaign was commenced, aiming to cover 34.4 million children nationwide, with an additional catch-up day for those missed during the campaign. Furthermore, UNICEF announced another drive for polio vaccination at the end of August.^6^ UNICEF also contributes by working on the border between Pakistan and Afghanistan to vaccinate the moving population.^7^

These efforts to contain the spread of polio were accompanied by thorough training of staff of the vaccination drive regarding strict adherence to prevent transmission of COVID-19. To operationalize these drives, infection prevention, and control equipment were also distributed that included thermometers, hand sanitizers, and masks.^8^ Revised operational modalities were determined during the August campaign and were put into effect in the scheduled September campaign (September 21–25). These modalities included revised team structures during the campaign and the use of more simplified logbooks. Recording, reporting, and marking of all the doors of the households were also resumed to aid monitoring and improve supervision. The September campaign aimed to vaccinate 40 million children younger than 5 years across the country.^9^ These three successful campaigns despite the COVID-19 scenario signify the paramount importance that Pakistan has given to the efforts in pursuit to eradicate polio.

## CHALLENGES AT HAND

To curb the spread of COVID-19 in Pakistan, a lockdown was imposed on March 23, 2020, which added to the already existing challenges in the polio immunization drives. On comparing the data of child polio immunization before lockdown (September 23, 2019–March 22, 2020) with the first 6 weeks of lockdown (March 23–May 9, 2020), it was noticed that the mean number of daily immunization visits decreased by 52.8% (from 5,184 to 2,450 visits) and only 92,492 children were immunized as compared with 608,832 children in the same period before lockdown.^10^

The polio immunization campaign in Pakistan is encountering numerous challenges resulting from inadequate health facilities, administrative and organizational deficiencies, and significant gaps in coverage due to refusals, inaccessibility of subjects, insecurity, and conflict.

In 2019, the WHO Technical Advisory Group on polio eradication reported that the “Pakistan program is on a failing trajectory, putting the rest of the global program at risk”.^11^ In 2018, GPEI stated that no significant improvement was seen in Pakistan’s eradication program since 2017. A strong correlation was found between unfortunate socioeconomic markers and the lack of poliovirus immunization. Shortage in sustenance and job opportunities co-existed with inadequate health facilities in certain at-risk communities in Pakistan.^12^ In addition, the estimation of the number of children requiring vaccines is hindered by a lack of electronic immunization records at some vaccination centers. Maintenance of a cold chain has also been problematic because of power shortage in Pakistan.^13,14^ Differences in population density across the country and inaccessible geographical locations in the south and the Himalayas act as barriers to polio immunization.^15^ Certain locations are known to be critical because of lack of vaccination services owing to alarming high crime rates, conflict, and insecurity. Global Polio Eradication Initiative has also reported cases of harassment of health workers, particularly female frontline workers.^12^ A noticeable discrepancy in immunization coverage has been seen at the regional and district levels; coverage is reported to be 75% in Punjab, whereas < 45% in Fata and Baluchistan provinces.^16^

Lack of knowledge and misconceptions regarding the polio vaccine, along with cultural, religious, and political barriers have collectively contributed to vaccine hesitancy and refusal among various communities across Pakistan.^12^ Spread of rumors and anti-polio sentiments, through social media, including the content of oral polio vaccine (OPV) (rumored to contain pork substances) and the effect of OPV on fertility, has led to strident refusals from parents to vaccinate their children.^17^ The current pandemic is now mainly affecting some of the poorest regions of Pakistan where there is limited access to proper health and hygiene needs, leading to both increased COVID-19 and polio cases.^4,18^

Recently, an analysis in August 2020 on the environmental sample detection of poliovirus in sewage specimens revealed 60% of positivity in samples as compared with 43% in August 2019.^8^ This shows a significant increase over the last year and is a major concern. Hence, efforts for immunization should be continued to contain the possible spread from the environment.^8^

## CONCLUSION

Efforts to curb the spread of polio amid the COVID-19 pandemic deserve attention as two competing health priorities come into play. Pakistan has been facing many challenges in past in the battle against polio, including lack of immunization services, vaccine misconceptions, and a fragile healthcare system to count a few. Lockdown and the added challenges due to COVID-19 have added insult to injury. Although successful efforts have been put into action to mitigate impacts on polio immunization with three successful immunization drives so far, there are still formidable challenges that are complicating nationwide immunization. Pakistan managed to flatten the COVID-19 curve in most of July till October that made three immunization drives possible, but the cases are on the rise again which can complicate the polio situation in the country if left unmonitored. Only Pakistan and Afghanistan remain endemic to this disease, and the challenges being faced by these countries deserve to be highlighted. The efforts in these two countries should be given the required impetus, so the whole world can be declared polio free.
